# Adult sacrococcygeal teratoma excised by endoscopic surgery with a transsacral approach: a case report

**DOI:** 10.1186/s40792-021-01263-y

**Published:** 2021-08-06

**Authors:** Ryosuke Machi, Chikashi Hiranuma, Hayato Suzuki, Masakazu Hattori, Kenji Doden, Yasuo Hashidume

**Affiliations:** 1 Department of Surgery, Fukui Prefectural Hospital, 2‑8‑1, Yotsui, Fukui, Fukui 910‑8526 Japan; 2 Department of Surgery, Keiju Medical Center, 94, Tomioka, Nanao, Ishikawa 926‑8605 Japan

**Keywords:** Adult, Sacrococcygeal teratoma, Endoscopic surgery, Transsacral approach

## Abstract

**Background:**

Although sacrococcygeal teratoma (SCT) is relatively common in children, it is rare in adults. The prognosis for malignant cases is poor, so prompt surgical resection is required. Transabdominal and transsacral approaches are common approaches for tumor resection. In recent years, there have been reports of tumor removal with laparoscopic assistance, but all have applied transabdominal approaches.

**Case presentation:**

A 27-year-old woman visited our gynecology department because of abdominal pain and genital bleeding. Magnetic resonance imaging (MRI) revealed a 3-cm-sized cystic mass in the left retrorectal area, and she was referred to our department for detailed examinations and treatment. She was diagnosed with a presacral cystic tumor and decided to undergo surgery. We used a transsacral approach to perform tumor excision. Since it was difficult to confirm the deep part of the tumor through direct visualization, we used GelPOINT® Path (a transanal access platform) and AirSEAL® System (insufflation device) to remove the tumor endoscopically. The postoperative course was uneventful with no bladder or rectal dysfunction. Histopathological examination revealed a mature teratoma.

**Conclusions:**

When the tumor is relatively small and located in the lower sacrum, the endoscopically assisted transsacral approach can establish a stable field of view by expanding the depth of the surgical field. This method is useful considering its ability to perform excision without leaving residual tumor tissue and satisfactory safety and cosmetic results.

## Background

Jackman et al. [1] classified anterior sacral masses into five categories: congenital, inflammatory, neurogenic, osteogenic, and others; most of these masses are congenital [2]. The anterior sacrum is the area surrounded by the rectum on the anterior surface, the sacral bone on the posterior surface, the peritoneal inversion on the upper surface, the levator ani and coccygeus muscles on the lower surface, and the rectal support tissue on the lateral surface. Since a caudal end forms here during the fetal period and many fetal tissues gather here, various congenital tumors may develop at this site, including teratomas.

Although sacrococcygeal teratoma (SCT) is relatively common in children, it is rare in adults. Since the prognosis for malignant cases is poor, prompt surgical resection is required [3].

Here, we present a case of an adult-onset presacral mature teratoma excised by endoscopic surgery using a transsacral approach.

## Case presentation

**Fig. 1:**
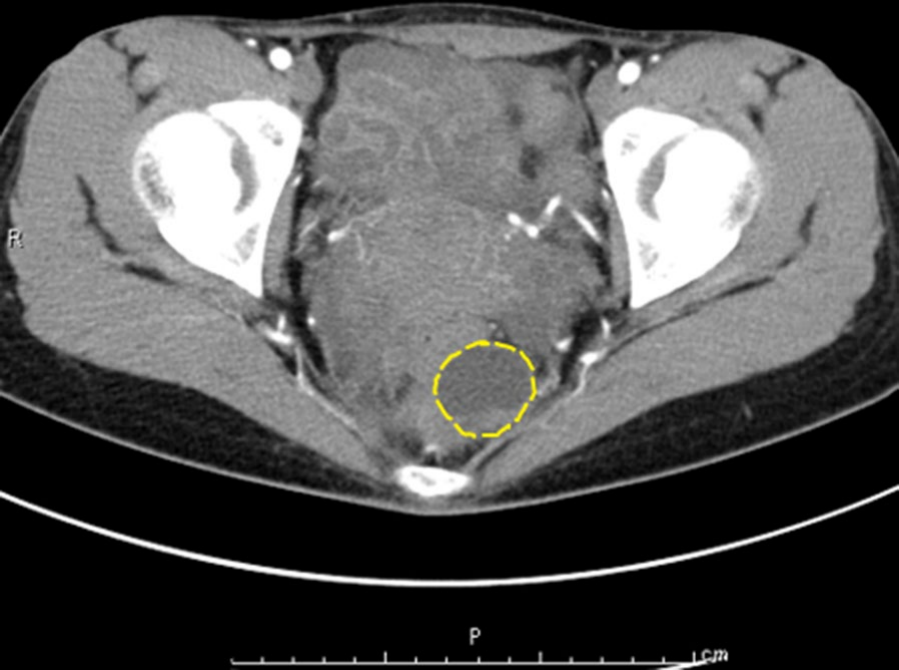
CT showed a multilocular cystic mass on the dorsal side of the rectum in front of the sacrum (dotted line)

**Fig. 2:**
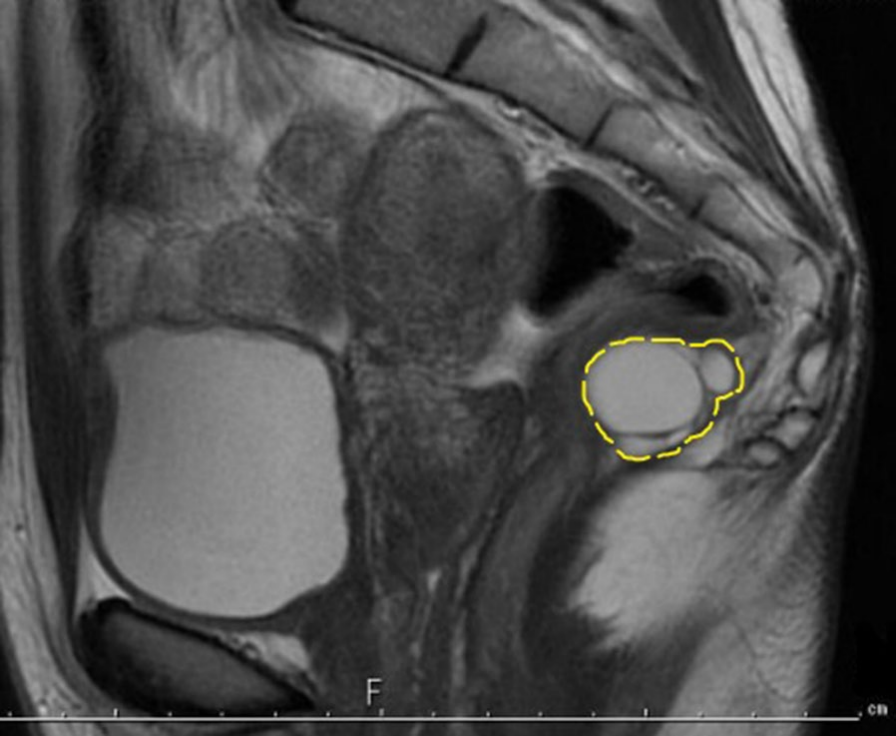
The tumor had a clear, smooth border and showed high signal intensity on T2-weighted images (dotted line). No fat was found in the tumor

**Fig. 3:**
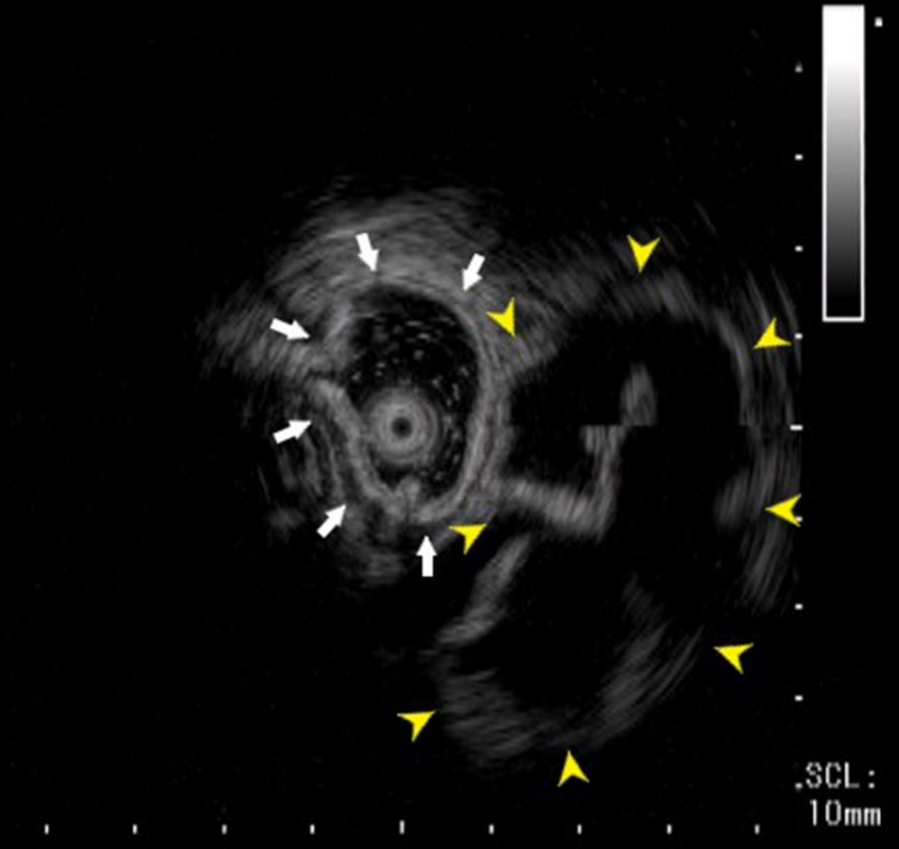
EUS showed a cyst (yellow arrowheads) outside the intestinal tract (white arrows). The cyst had a uniform wall with septa, showed no obvious irregular nodules or solid components, and had no internal calcification

A 27-year-old woman visited our gynecology department because of abdominal pain and genital bleeding. Magnetic resonance imaging (MRI) revealed a cystic mass in the left retrorectal area, and she was referred to our department for detailed examinations and treatment. Her medical history was unremarkable. Her laboratory results, including serum tumor biomarkers, such as α-fetoprotein (AFP), carcinogenic embryonic antigen (CEA) and carbohydrate antigen 19-9 (CA19-9), were within normal limits. Computed tomography (CT) showed a 3-cm-sized multilocular cystic mass in the anterior sacrum on the left dorsal side of the rectum. The mass showed a gradual contrast effect on the margin and was suspected to have a solid component. No obvious calcification was observed (Fig. 1). MRI showed no obvious fat in the area (Fig. 2). Fluorodeoxyglucose-position emission tomography (FDG-PET) showed no abnormal uptake. Lower gastrointestinal endoscopy showed no abnormalities in the rectal mucosa. Endoscopic ultrasound (EUS) showed a 3-cm-sized extraintestinal cyst near the rectum. No obvious calcification was found inside (Fig. 3).

With the above tests, it was difficult to confirm the preoperative diagnosis of the presacral cystic mass. Teratoma, tailgut cyst, dermoid cyst, epidermoid cyst, etc., were considered in the differential diagnosis. Because there have been reports of malignant cases, we decided to perform surgical mass removal as a diagnostic treatment.

**Fig. 4:**
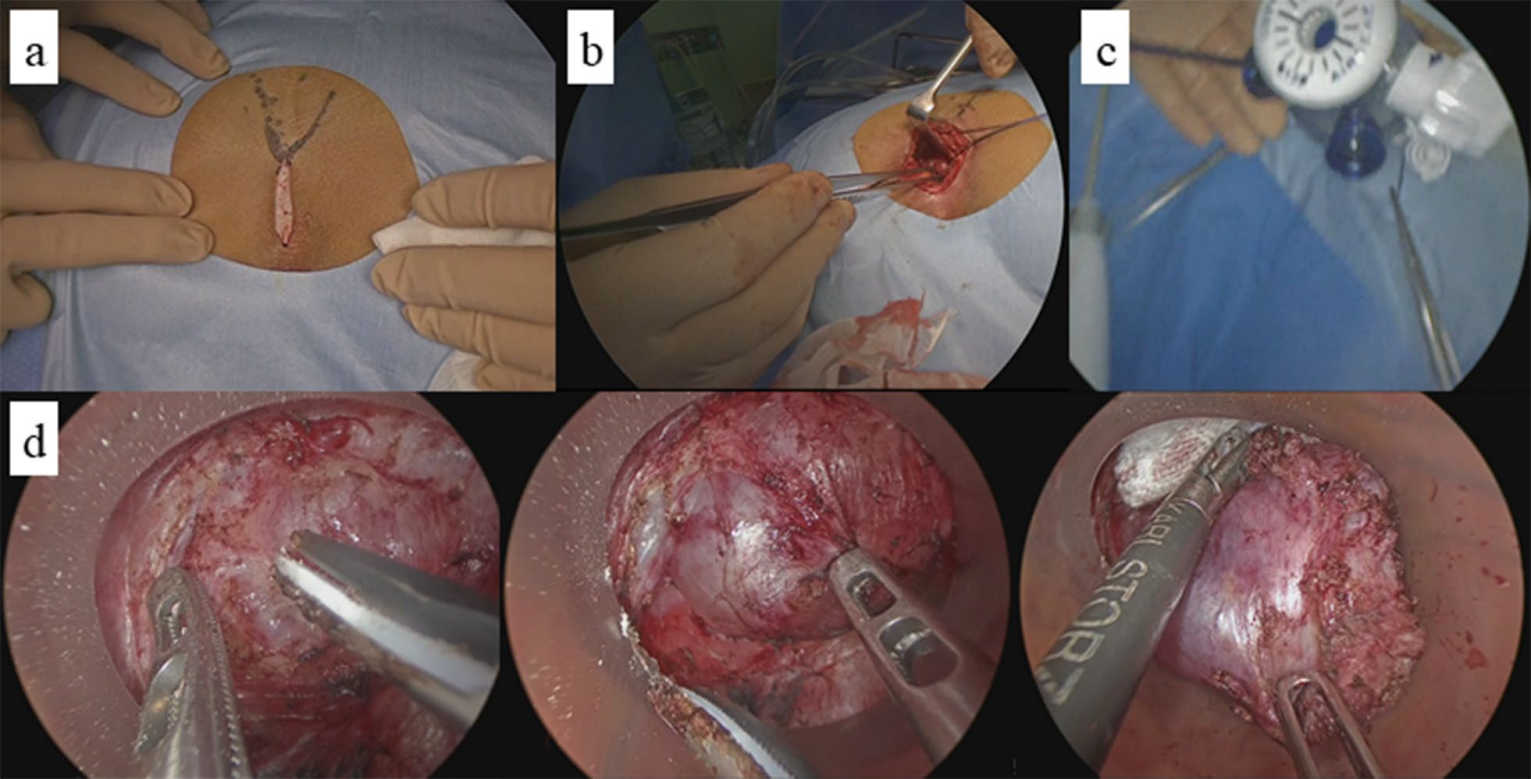
A skin incision measuring approximately 3 cm was made from the coccyx to the anus (**a**). It was difficult to confirm the deep part of the tumor through direct visualization (**b**). The tumor was resected endoscopically using the GelPOINT® Path and AirSEAL® System (**c**, **d**)

The operation was conducted with the patient in the jack-knife position. A skin incision measuring approximately 3 cm was made from the coccyx to the anus. We made an incision in the mural fascia to reach the tumor and enable detachment without damaging the tumor. Since it was difficult to confirm the deep part of the tumor through direct visualization, we attached a GelPOINT® Path (Applied Medical, USA) placed two 10-mm trocars and a AirSEAL® (CONMED, USA) trocar in a triangular fashion to the incision and insufflated it at 12 mmHg with the AirSEAL® System to remove the tumor endoscopically while confirming the boundary between the tumor and the anterior coccyx/posterior rectal wall. The operative time was 171 min, with minimal blood loss (Fig. 4).

**Fig. 5:**
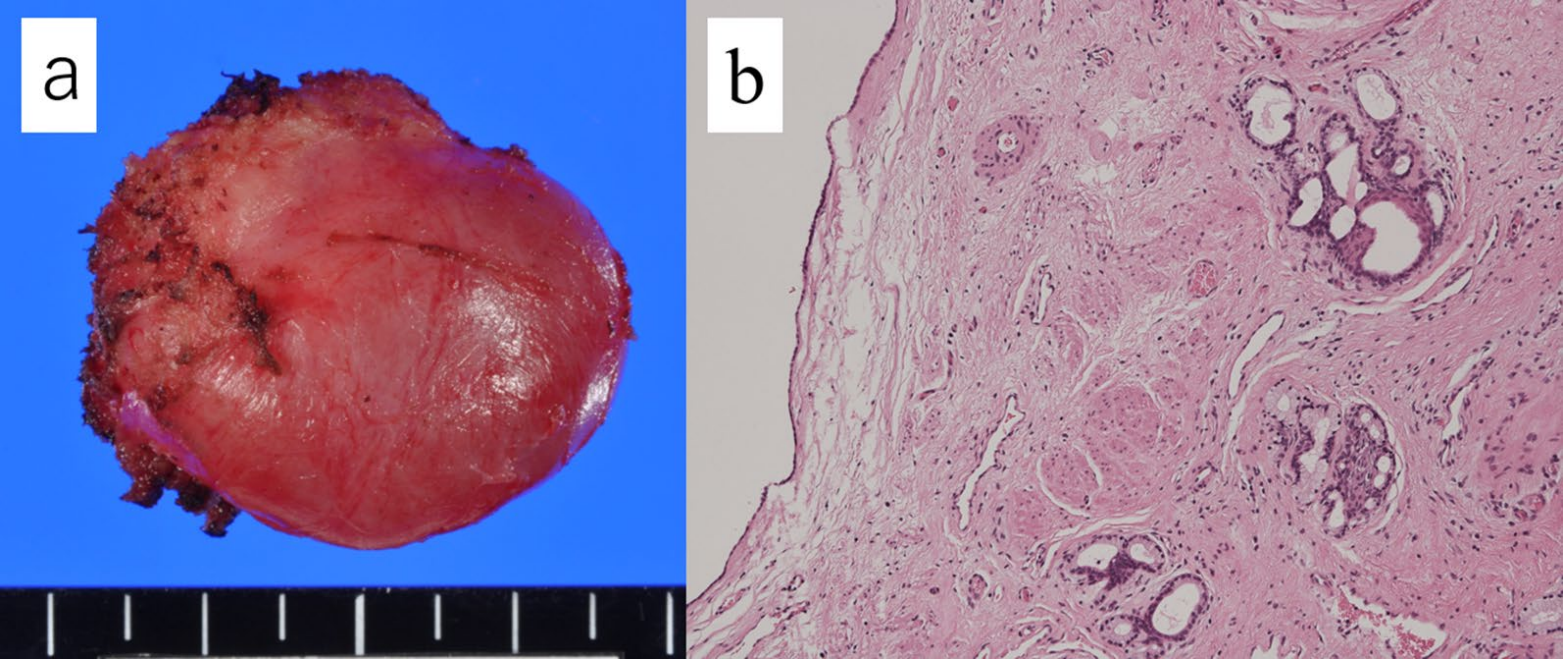
A multilocular tumor measuring 3 cm in diameter (**a**). The cyst wall was covered with hairline epithelium, stratified squamous epithelium, and columnar epithelium, and the cyst stroma had fibrous tissue, smooth muscle tissue, and peripheral nervous tissue (**b**: hematoxylin and eosin (HE) staining × 40)

Histopathological examination revealed a benign mature teratoma (Fig. 5). She did not have any complications and was discharged from our hospital 5 days after the surgery. Seven months later, she had no tumor recurrence or residual symptoms.

## Discussion

Teratomas are mixed tumors that contain two or more of the three germ layer components. These most often occur in the gonads and in the mediastinum, retroperitoneum, sacrococcygeal region, and central nervous system. The incidence of SCTs is reported to be 1 in 35,000 to 1 in 40,000 live births, with a 4:1 female predominance [4]. According to the Altman classification [5], SCTs can be divided into four types: type I, entirely outside the pelvis; type II, mostly outside the pelvis but with a small intrapelvic component; type III, mostly inside the pelvis, with a small external mass; and type IV, entirely inside the pelvis. Most type I and type II SCTs, which are easy to find, are diagnosed and resected in early childhood, but the reason why there are a small number of reports of adult cases is thought to be because most adult SCTs are type III and type IV, which are difficult to find. Most SCTs are benign, with only 1–2% showing malignant transformation, but the prognosis of malignant SCTs is poor, so prompt surgical resection is required [3, 6]. Taking into account the risk of recurrence or dissemination in cases of malignancy, preoperative pathological studies such as transcutaneous or transrectal biopsy should be avoided [7].

Complete surgical excision is the most acceptable treatment for SCT, and the main surgical approaches generally include transabdominal, transsacral, or a combination of both. The transabdominal approach is typically selected for high lesions and provides good exposure of the tumor posterior to the rectum. The transsacral approach is preferred for small tumors located in the lower portion of the pelvis. Although there are no clear selection criteria, the transsacral approach is considered to be a good option for tumors with a diameter of 10 cm or less and localization below the third sacral vertebra (S3) [2, 8]. Recent reports have documented that some SCTs can be safely excised laparoscopically, but in all cases, the transabdominal approach was applied [7, 9–11]. To the best of our knowledge, this is the first case report of a successful excision of an SCT by endoscopic surgery using a transsacral approach. This case featured a young woman who requested the skin incision to be as small as possible in consideration of esthetic and cosmetic results, and the tumor was 10 cm or less in diameter and below S3. Therefore, we chose a transsacral approach with endoscopic assistance. In the operation, the skin incision was limited to approximately 3 cm, and when it was difficult to remove the deep portion of the tumor through direct visualization, we attached GelPOINT® Path to the incision and insufflated it with the AirSEAL® System to excise the tumor endoscopically. We were able to excise the tumor without damaging the tumor or leaving residual tissue and did not cause complications, such as bladder and rectal dysfunction. This method is considered to be useful because the skin incision can be minimized and a safe operation can be performed with a stable field of view by ensuring a deep surgical field. However, if the patient has a large tumor or high lesion, careful judgment is required before selecting this procedure.

## Conclusions

Small SCTs located in the lower sacrum can be safely excised by endoscopic surgery using a transsacral approach.

This surgical procedure is useful in terms of its ability to perform excision without leaving residual tumor tissue and satisfactory safety and cosmetic results.

## Data Availability

All data generated during this study are included in this published article.

## Abbreviations

SCT: Sacrococcygeal teratoma
MRI: Magnetic resonance imaging
AFP: α-Fetoprotein
CEA: Carcinogenic embryonic antigen
CA19-9: Carbohydrate antigen 19-9
CT: Computed tomography
FDG-PET: Fluorodeoxyglucose-position emission tomography
EUS: Endoscopic ultrasound
